# Ensiling Grape Pomace With and Without Addition of a *Lactiplantibacillus plantarum* Strain: Effect on Polyphenols and Microbiological Characteristics, *in vitro* Nutrient Apparent Digestibility, and Gas Emission

**DOI:** 10.3389/fvets.2022.808293

**Published:** 2022-02-24

**Authors:** Palmira De Bellis, Aristide Maggiolino, Clara Albano, Pasquale De Palo, Federica Blando

**Affiliations:** ^1^Institute of Sciences of Food Production (ISPA), National Research Council (CNR), Bari, Italy; ^2^Department of Veterinary Medicine, University of Bari “Aldo Moro”, Bari, Italy; ^3^Institute of Sciences of Food Production (ISPA), National Research Council (CNR), Lecce, Italy

**Keywords:** grape pomace, silage, polyphenol, antioxidant capacity, lactic acid bacteria, *in vitro* digestion

## Abstract

The present study investigated the effects of different grape pomace storage techniques on the effectiveness as feed on *in vitro* ruminant digestion efficiency. Grape pomace from an autochthonous red grape variety (cv Nero di Troia) was used as fresh (GP) or ensiled, both without additives (SIL) and with the addition of a bacterial strain, *Lactiplantibacillus plantarum* 5BG (SIL+). All the different storage treatments were subject to chemical and microbiological evaluation, as well as *in vitro* digestibility, and gas production. Microbiological data revealed the good quality of grape pomace and silages due to the lactic acid bacteria populations and low presence, or absence, of undesirable microorganisms. The addition of *L. plantarum* 5BG influenced the chemical characteristics of the silage (SIL+). Ensiling technique deeply changed the polyphenolic composition, reducing anthocyanins, flavonols, and flavanols (condensed tannins precursors), particularly when *L. plantarum* 5BG was added. Antioxidant capacity was reduced by ensiling, in correlation with the polyphenolic content decrease. The oxygen radical absorbance capacity (ORAC) value of SIL+ was the lowest (*P* < 0.01) and its total phenol content was lower than SIL (*P* < 0.01). No statistical differences were observed between GP, SIL, and SIL+ on the antioxidant capacity by TEAC assay (*P* > 0.05). Ensiling did not affect the grape pomace nutrient profile, except for the reduction in NFC content. Apparent *in vitro* digestibility showed how ensiling increased dry matter (DM), organic matter (OM), neutral detergent fiber (NDF), crude protein (CP), ether extract (EE), and non-fiber carbohydrates (NFC) disappearance (*P* < 0.01), particularly with the *L. plantarum* 5BG inoculation. Moreover, SIL+ showed the lowest propionic acid (*P* < 0.05) and the highest methane (*P* < 0.01), butyric acid (*P* < 0.01), and nitrogen (*P* < 0.05) *in vitro* production. Ensiling GP resulted in a better *in vitro* digestibility, particularly if *L. plantarum* 5BG strain is added, probably due to the reduction of flavanols and their lower microbial activity inhibition.

## Introduction

The use of agricultural by-products as animal dietary supplement is the result of multiple efforts in reducing the carbon footprint of the livestock industry (1, 2). By-products are generally inexpensive and their production can be considered free of greenhouse gas emissions as they are allocated to the primary product (3). This leads to an increased interest in exploiting plant products and by-products as feed additives to solve both animal nutrition problems and livestock production impact. In fact, plant by-products are often rich in readily fermentable carbohydrates and fats, useful for animal digestion processes, but also in plant secondary compounds that have been reported to suppress CH_4_ concentration, reduce rumen protozoa counts, and modulate rumen fermentation patterns (4).

Grape pomace (GP) is the by-product of a winemaking process and consists of pressed grapes (skin and seeds with residual pulp) and stalk residues. Considering that the winemaking process does not allow a complete extraction of polyphenolic compounds, GP represents an abundant and inexpensive polyphenols source. In particular, GP is rich of flavonoids, the largest group of polyphenols predominantly found in skins, seeds, and stems (5). Polyphenols have been intensively studied for their anti-inflammatory and anti-microbial properties and for their health-associated effects against chronic diseases, such as cancer, neurodegeneration, and cardiovascular pathologies (6). The health benefits of GP polyphenols, especially flavonoids, have received great interest of researchers, and the food and nutraceutical industries (7). Grape pomace also might be considered an important feed ingredient in ruminants' diet, being rich in bioactive polyphenols and soluble fiber, particularly when climatic conditions limit the availability of other feeds (8, 9).

In contrast to humans, in farm animals the potential health-promoting effects of bioactive polyphenols have been considered only recently (5, 9, 10). The use of GP is suggested as an alternative ingredient in animal feeding, effective in enhancing the oxidative stability of the meat (inhibiting the meat lipid peroxidation), in reducing the addition of synthetic antioxidant as vitamin E, and in modulating the intestinal microbiota (increasing the presence of specific beneficial bacteria strains) (5). Moreover, the addition of GP to dairy cows' feed was found to modulate milk's fatty acid composition and methane emissions (11–13). So, when shortage of feeds occurs, the addition of GP could represent an alternative feed resource in ruminants (14).

Although GP can be used fresh as a feeding ingredient, the seasonality in its production combined to not suitable storage techniques can lead to nutrient losses and spoilage processes (8). Ensiling is an appropriate method to preserve this product along the time, allowing farmers to use it continuatively in the year. Moreover, the use of suitable bacterial inoculants could improve the quality and aerobic stability of silages (15–17). Most of the inoculants used belong to lactic acid bacteria (LAB) (15–20). During the ensiling process, LAB converts water-soluble carbohydrates (WSC) into organic acids (e.g., lactic acid and acetic acid) which rapidly reduce the silage pH. The acidic and anaerobic conditions help to inhibit the proliferation of undesirable microorganisms (e.g., Clostridia) and preserve nutrient components (19). Furthermore, LAB is known to degrade phenolic compounds (21), but few studies have focused on the effects of LAB inoculants on changes of phenolic compounds in silages (19) and on silage digestibility. Considering the results of previous publications, it could be hypothesized that ensiling GP could improve its nutritive characteristics as animal feeding and that the addition of starter strains could be useful both to improve its digestibility as well as its impact on rumen gas production. Therefore, the aim of this research was to evaluate the effects of ensiling with and without addition of *Lactiplantibacillus plantarum* 5BG (previously named *Lactobacillus plantarum*) (22) on the chemical composition, in particular polyphenolic compounds, microbiological, and nutritional quality of GP silages.

## Materials and Methods

The protocol for animal research was approved by the Ethics Committee for animal testing–CESA (process number 2-X/17) of the Department of Veterinary Medicine of the University of Bari “Aldo Moro”, Bari, Italy.

### Preparation of Silage Grape Pomace

Fresh grape pomace samples (*Vitis vinifera* L., cv Nero di Troia) from red cultivars were collected in the Puglia Region (South Italy), immediately after the crush of grape juice and fermentation processes for red wine production had been completed. The whole sampled grape pomace was randomly subdivided in three experimental treatments: (A) fresh grape pomace (GP); (B) silage (SIL); and (C) silage inoculated with *L. plantarum* 5BG (SIL+).

For silage, grape pomace was randomly assigned to SIL or SIL+, and 8 silos for each experimental group were prepared. Fresh GP was subdivided in 8 parts of similar volume of each silo. Silos were a cylindric plastic container (35 L vol; about 30 cm diameter ×50 cm high). Each silo was filled with manually pressed pomace. In the SIL+ group, inoculation of GP was performed with the strain *L. plantarum* 5BG (23) belonging to the Culture Collection of the Institute of Sciences of Food Production, National Research Council (ITEM 17403, http://server.ispa.cnr.it/ITEM/Collection/). A freeze-dried powder of *L. plantarum* 5BG was inoculated in GP at a final concentration of ~6 log cfu/g of GP. All the silos had a gas release valve in the lid and were stored upright at ambient temperature (18–25°C). The silage density was calculated as the ratio between the ensiled GP and silo volume (kg GP m^3^) and it was on average value of 658.54 ± 16.20 kg GP m^3^. The silos were opened after 1 month from the starting of the experiment (T0).

Fresh (GP) and ensiled grape pomace (SIL and SIL+) were subjected to the evaluation of pH, chemical and microbiological characteristics, *in vitro* digestibility, and gas production.

All samples (GP, SIL, SIL+) were stored lyophilized (Labconco Corp., Kansas City, MO) at −20°C until polyphenolic analysis. Afterward samples (GP, SIL, and SIL+) were ground using a CT 193 Cyclotec mill fitted with 1-mm screen (FOSS, Hilleroed, Denmark), and analyzed for phenols and antioxidant capacity at least in triplicate.

### Chemical Analyses

Before and after *in vitro* digestion, all GP, SIL, and SIL+ samples were analyzed in triplicate, considering each silo as the experimental unit. Dry matter (DM) was determined using standard procedures (24) (method 930.15). Ash was determined by standard procedures (24) (method 942.05) using a muffle furnace at 550°C for 16 h. Fat was determined using the Soxhlet extraction procedure (24) (Method 991.36), crude protein (CP) was determined by Kjeldahl N ×6.25 procedures (24) (Method 968.06). Neutral detergent fiber (NDF) and acid detergent fiber (ADF) were determined with the ANKOM fiber analyzer according to Van Soest et al. (25) and was corrected for residual acid-insoluble ash. Sodium sulfite was added to the solution for NDF determination. Non-fiber carbohydrates (NFC) were calculated by subtracting CP, ether extract (EE), and NDF from the organic matter (OM) and the metabolizable energy (ME) values were calculated using the equation:


$$\begin{array}{l} ME\;(MJ/kg\;DM)=0.82\;\times \;[(2.4\;\times \;CP)\;+\;(3.9\;\times \;EE)\;+\\ \;(1.8\;\times \;OM\;residual)\;\times \;OM\;digestibility] \end{array}$$

as suggested by Robinson et al. (26), where CP, EE, and OM residual are as g/kg of DM. The water soluble carbohydrate (WSC) content was determined as described by McDonald et al. (27).

### Microbiological Analyses

Microbiological analyses were carried out on SIL and SIL+ at different times (0, 24, 48, 144 h) after silos opening, as well as on GP. Twenty grams of samples were homogenized with 180 mL of sterile buffered peptone water (BPW, Biolife) in a Stomacher (Seward, London, United Kingdom) for 2 min. The resulting suspensions were serially diluted in the same diluent and plated in duplicate on the following agar media for the detection and enumeration of microorganisms: Plate Count Agar (PCA, Difco, Franklin Lakes, NJ) supplemented with 100 mg/L of cycloheximide (EMD Millipore Corp., Billerica, MA) incubated at 30°C for 24 h to determine the total aerobic mesophilic bacterial counts (AMB); de Man Rogosa Sharpe (MRS) agar (Oxoid Ltd, Basingstoke, UK) supplemented with 100 mg/l of cycloheximide incubated at 30°C for 48 h for the determination of LAB; Sabouraud Dextrose Agar (SDA, Oxoid) supplemented with 200 mg/L chloramphenicol (Sigma, Milan, Italy) for the enumeration of yeasts and molds, incubated at 25°C for 72 h; violet red bile glucose agar (VRBGA, Difco) for total *Enterobacteriaceae*, incubated aerobically at 37°C for 24 h; sulfite polymyxin sulfadiazine (SPS) agar (Biolife, Milan, Italy) for detecting *Clostridium perfringens*, incubated anaerobically at 37°C for 24 h; the chromogenic and selective *Listeria* agar (ALOA, Biolife) for the enumeration of *Listeria monocytogenes* after 48 h of incubation at 37°C. Moreover, an aliquot of each microbial suspension was heat-treated for 20 min at 90°C, plated on a plate count agar (PCA, Difco) and incubated for 24 h at 30°C for spore-forming bacteria (SFB) counts. LAB, AMB, yeasts, molds, and *Enterobacteriaceae* counts were used as indicators of the overall microbiological quality of samples, while SFB, *C. perfringens*, and *Ls. monocytogenes* were also considered as potential pathogens and indicators of the product microbiological safety (28). Each sample was tested in triplicate.

### Preparation of Polyphenolic Extracts From Grape Pomace

Polyphenols were extracted in triplicate from 500 mg (DW, dry weight) GP, SIL, and SIL+ after silos opening (T0), macerated with 50 mL extraction solvent (70% acetone + 0.01% TFA), at 4°C, over-night. After centrifugation of the slurry (10 min at 2,000 × *g*) the supernatant was collected, further 10 mL of extraction solvent were added to the pellet, and the extraction was repeated on a rotary shaker for 1 h. Pooled supernatants were evaporated *in vacuo* at 32°C using a model R-205 Büchi rotavapor (Büchi Labortechnik AG, Switzerland) and re-suspended in acidified water (0.01% trifluoracetic acid) (TFA) at known volume. Extracts were filtered on 0.45 μm CA syringe filter (Filtres Fioroni, France), portioned and stored at −20°C until analysis. The extraction experiments were performed twice, with each triplicated extraction considered for HPLC injection.

### Identification and Quantification of Polyphenolic Compounds

The HPLC separation, identification, and quantification of polyphenols in GP, SIL, and SIL+ extracts were performed using the same chromatographic method and column as already reported (29). The polyphenolic compounds were identified by comparing their peak retention times and UV-visible spectra with those of commercial standards, where available. Spiking experiments using sample solutions and standards as well as comparisons to any relevant published grape pomace analytical characterization were used to verify peak identities (30).

The identified phenolic compounds were quantified by the external standard method using a six-points calibration curve of oenin (1.25–250 mg/L), gallic acid (1–100 mg/L), syringic acid (0.5–100 mg/L), catechin (1–100 mg/L), and rutin (0.5–100 mg/L). When reference standard compounds were not available, the quantification was done using the calibration curve of the most structurally related substance, including a molecular correction factor (31).

### Phenols and Hydrophilic Antioxidant Capacity

The polyphenolic extracts were assessed for total phenol content and reducing capacity by the Folin-Ciocalteu (F-C) assay, as well as their antioxidant capacity using the ABTS assay (Trolox-equivalent antioxidant capacity - TEAC) and the oxygen radical absorbance capacity (ORAC) assay (29). A rapid microplate methodology, using a microplate reader (Infinite M-200, Tecan Group Ltd, Männedorf, Switzerland) and 96-well plates (Costar, 96-well clear round bottom plate, Corning) was used.

Two independent plates (at least) were performed for each sample, which was tested in triplicate for each dilution (four dilutions of each extract).

### *In vitro* Rumen Digestion

All the operations of rumen fluid sampling were performed at the slaughterhouse, suddenly after animal slaughtering. Rumen fluid was obtained by a total of 24 animals (Limousine steers, aged 12 months, reared in the same farm). All animals were fed *ad libitum* with the same feed ratio (86.7% of dry matter, 15.9% of crude protein, 9.2% of crude fiber, 25.6% of NDF, 10.1% of ADF, and 2.6% of ADL). Samples of ruminal contents (filtered through eight layers of gauze cloth) were collected in thermos flasks (previously filled with distilled water at 39°C to avoid thermal shock to the rumen fluid), insufflating CO_2_ into the headspace to ensure the environment remained anaerobic and taken within 30 min to the laboratory. After transport, the top layer of ruminal contents was discarded, and the remaining portion was mixed and blended under a CO_2_ headspace for 1 min to remove any additional particles and/or attached organisms. The combined fluid and contents were strained through 6 layers of cheesecloth to form the inoculum for the *in vitro* fermentation (32).

*In vitro* fermentation was conducted for 48 h using the Daisy II incubator system (ANKOM Tech., Fairport, NY), as described by Maggiolino et al. (32). The unit consisted of four incubation vessels with a capacity of 2 L for each. Each vessel contained 1.6 L of buffer solution, 400 mL of rumen liquor, and 25 nylon filter bags (ANKOM F57, ANKOM Tech., Fairport, NY). The buffer solution consisted of 1.33 L buffer A (KH_2_PO_4_, 10.0 g/L; MgSO_4_ H_2_O, 0.5 g/L; NaCl, 0.5 g/L; CaCl_2_ H_2_O, 0.1 g/L; and urea, 0.5 g/L) and 266 mL of buffer B (Na_2_CO_3_, 15.0 g/L and Na_2_S_7_H_2_O, 1.0 g/L), mixed in each digestion vessel and the pH was adjusted to 6.8, as reported by the method of Marten and Barnes (33). Each digestion trial for each sample was performed in duplicate. Bags were rinsed in acetone and allowed to air dry before drying at 100°C for 24 h, after recording dry bag weight. They were used and filled with a total of 500 mg each GP, SIL, and SIL+ (all samples were stored at −20°C before analysis). All samples had been previously ground until the particle size reached 2 mm screen using a hammer mill (Pullerisette 19, Fritsch GmbH, Laborgeratebau, Germany). Twenty-five bags were put in each incubation vessel. After digestion, all bags were weighed again. All analysis for nutrient parameters was performed after digestion and percentage of disappearance for each parameter was calculated as: (PB-PA)/PB, where PB is the quantity (g/kg) of parameter in the samples before the digestion and PA is the quantity (g/kg) of the parameter after digestion. Results were expressed as percentage.

### *In vitro* Gas Production and Analysis

For gas and volatile fatty acid analysis, an automated pressure transducer system (Ankom Technology, Macedon, NY) was used as described by Maggiolino et al. (32). It was equipped with 8 different 250 mL bottles. The same buffer solutions for the *in vitro* digestion section were used. Each vessel received 133.3 mL of Buffer A and 26.7 mL of Buffer B. Then, 40 mL of rumen fluid was added. The GP, SIL, and SIL+ (500 mg) were pre-weighed into each vessel. The head space of each vessel was insufflated with CO_2_ for 2 min to ensure anaerobic conditions.

Vessels were put in an oscillating water bath (39°C with an oscillating frequency of 45/min), to reproduce movements similar to those found in the rumen, and digestion was simulated for a 48-h fermentation period. After this period, vessels were removed from the water bath and placed into an ice bath while gas samples were drawn into evacuated test tubes, as described by Trotta et al. (34). Gas samples were analyzed for methane production with gas chromatography (Agilent Technologies, Santa Clara, CA) by using the total gas volume at standard temperature and pressure (35). Flasks were opened, pH measured, and 1 mL aliquot of the fermentation medium was combined in a 1.5 mL centrifuge tube with 0.1 mL of 500 g/L metaphosphoric acid and 0.1 mL of 85 mM of 2-ethyl butyrate. Samples were centrifuged at 39,000 × *g*, at 23°C for 15 min. Afterward, they were processed for volatile fatty acid (VFA) concentrations (35) using a gas chromatograph with FID (Agilent Technologies, Inc., Santa Clara, CA) equipped with a 2 m ×3 mm packed column (45.60 Carboxen 1000, Supelco, Inc., Bellefonte, PA). For determination of ammonia nitrogen, 2 mL fluid and 2 mL trichloro-acetic acid solution (10%, w/v) were mixed to deproteinize the samples and then centrifuged for 5 min at 1,500 × *g*. The supernatant (2 mL) was processed in order to measure the ammonia nitrogen concentration according to a spectrophotometric method (36).

### Statistical Analysis

One-way analysis of variance (ANOVA) was performed to differentiate among the treatments (GP, SIL, and SIL+), the phenolic content, the *in vitro* digestion data, and gas emission results. The ANOVA was performed using the general linear model (GLM) by SAS software (37), according to the following model:


$$\begin{array}{r} {\text{y}}_{\text{ij}}=\mu +\alpha_{\text{i}}+{\text{G}}_{\text{j}}+\varepsilon_{\text{ijk},} \end{array}$$

where y_ij_ represents all the previous cited patterns as dependent variables; μ is the overall mean; α_i_ is the silo random effect; G was the effect of the jth group (GP, SIL, and SIL+) (j = 1, …3) and ε_ijk_ was the error term.

The microbial data after ensiling were subject to a multifactorial ANOVA according to the following model:


$$\begin{array}{r} {\text{y}}_{\text{ijk}}=\mu +\alpha_{\text{i}}+{\text{G}}_{\text{j}}+{\text{T}}_{\text{k}}+{\left(\text{G}\times T\right)}_{\text{jk}}+\varepsilon_{\text{ijkl},} \end{array}$$

where y_ij_ represents all the microbial patterns as dependent variables; μ is the overall mean; α_i_ is the silo random effect; G was the effect of the jth group (SIL and SIL+) (j = 1, 2); T represents the effect of the kth time after desilation (k = 1, …4); G × T represents the effect of the binary interaction of the jth group and kth time (1,…,8) and ε_ijk_ was the error term. When not significant, the binary interaction was dropped from the model. A Tukey test was applied to evaluate the differences according to time.

The significance was set at *P* < 0.05, and the results were expressed as means and standard error of the means.

## Results

### Chemical Composition and Microbiological Analyses

The chemical composition of GP, SIL, and SIL+ is shown in Table 1. There are no differences after ensiling with or without *L. plantarum* 5BG in DM, CP, EE, NDF, ADF, and ash (*P* > 0.05), although ME showed the highest values in SIL+ (*P* < 0.01) and lower in SIL compared to GP (*P* < 0.05). NFC content was lower in SIL+ than GP and SIL (*P* < 0.01). Moreover, ensiling (both SIL and SIL+) showed lower pH compared to the GP (*P* < 0.001) and WSC disappeared (*P* < 0.001).

**Table 1:** Impact of ensiling with or without *L. plantarum* 5BG on chemical composition and pH of grape pomace.

	**GP**	**SIL**	**SIL+**	**MSE**	***P*-value**
DM (%)	42.6	43.9	43.9	0.211	0.0422
CP, % of DM	9.8	9.9	10	0.078	0.1699
EE, % of DM	5.1	4.8	4.8	0.063	0.2258
Ash, % of DM	6.9	7.3	7.4	0.081	0.2882
NDF, % of DM	67.4	68.2	68.8	0.237	0.3885
ADF, % of DM	35.1	34.5	35.5	0.117	0.6221
NFC, % of DM	14.0^A^	12.6^B^	10.4^C^	0.323	<0.0001
WSC, % of DM	0.025^A^	0.00^B^	0.00^B^	0.001	<0.0001
pH	4.82^A^	4.52^B^	4.49^C^	0.01	<0.0001
ME, Mj/kg DM	9.91^ab^	9.67^b^	10.10^a^	0.07	0.0411

*DM, dry matter; CP, crude protein; EE, ether extract; NDF, neutral detergent fiber; ADF, acid detergent fiber; NFC, non-fiber carbohydrate; WSC, water soluble carbohydrates; ME, metabolizable energy; GP, grape pomace; SIL, grape pomace after silage; SIL+, grape pomace after silage with L. plantarum 5BG; MSE, mean standard error*.

*Different letters correspond to statistical differences. A, B, C = P <0.01; a, b, c = P <0.05*.

**Figure 1:**
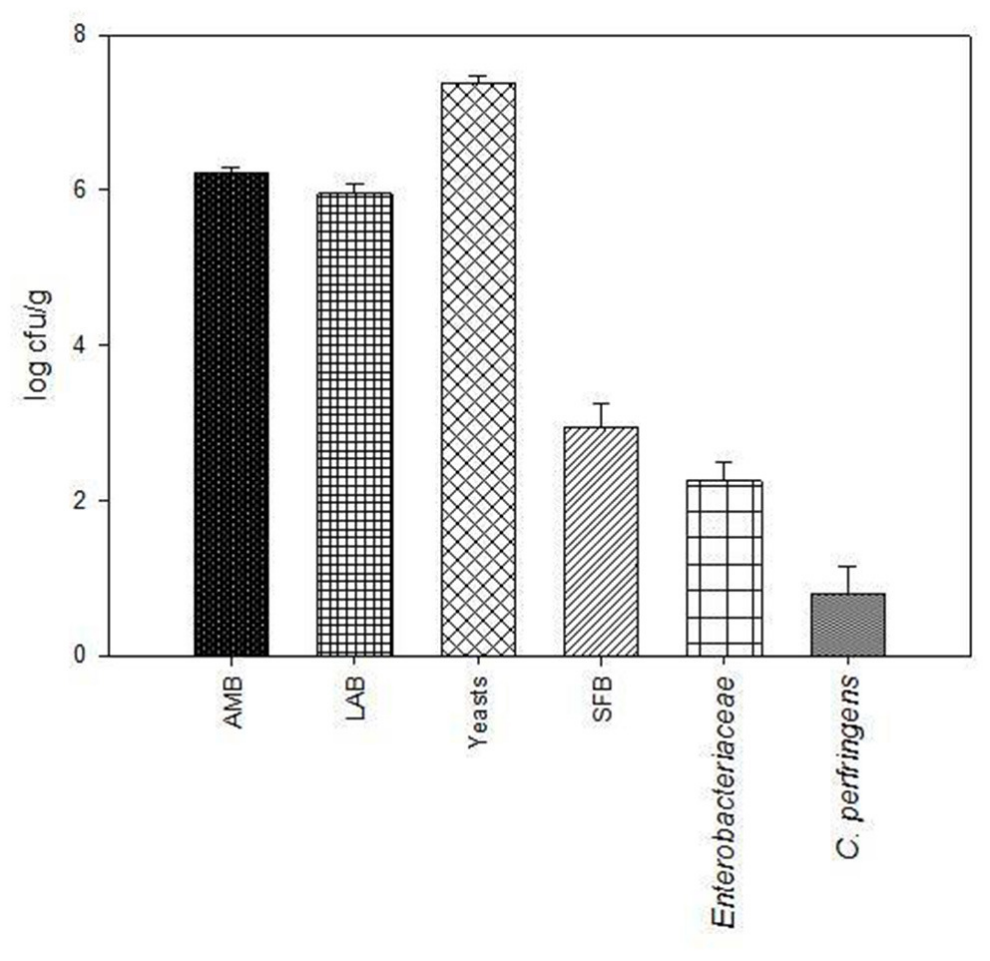
Microbiological characteristics of grape pomace (GP). The microbial loads (log cfu/g) of total aerobic mesophilic bacteria (AMB), lactic acid bacteria (LAB), yeasts, spore-forming bacteria (SFB), *Enterobacteriaceae*, and *C. perfringens* are reported as means and standard deviation (error bars).

The results of GP microbiological analyses, before ensiling, are shown in Figure 1. Total AMB and LAB loads were 6.22 ± 0.07 log cfu/g and 5.96 ± 0.13 log cfu/g, respectively. Yeast load was 7.39 ± 0.08 log cfu/g, while the cell densities of SFB, *Enterobacteriaceae*, and *C. perfringens* were 2.94, 2.26, and 0.8 log cfu/g, respectively.

**Figure 2:**
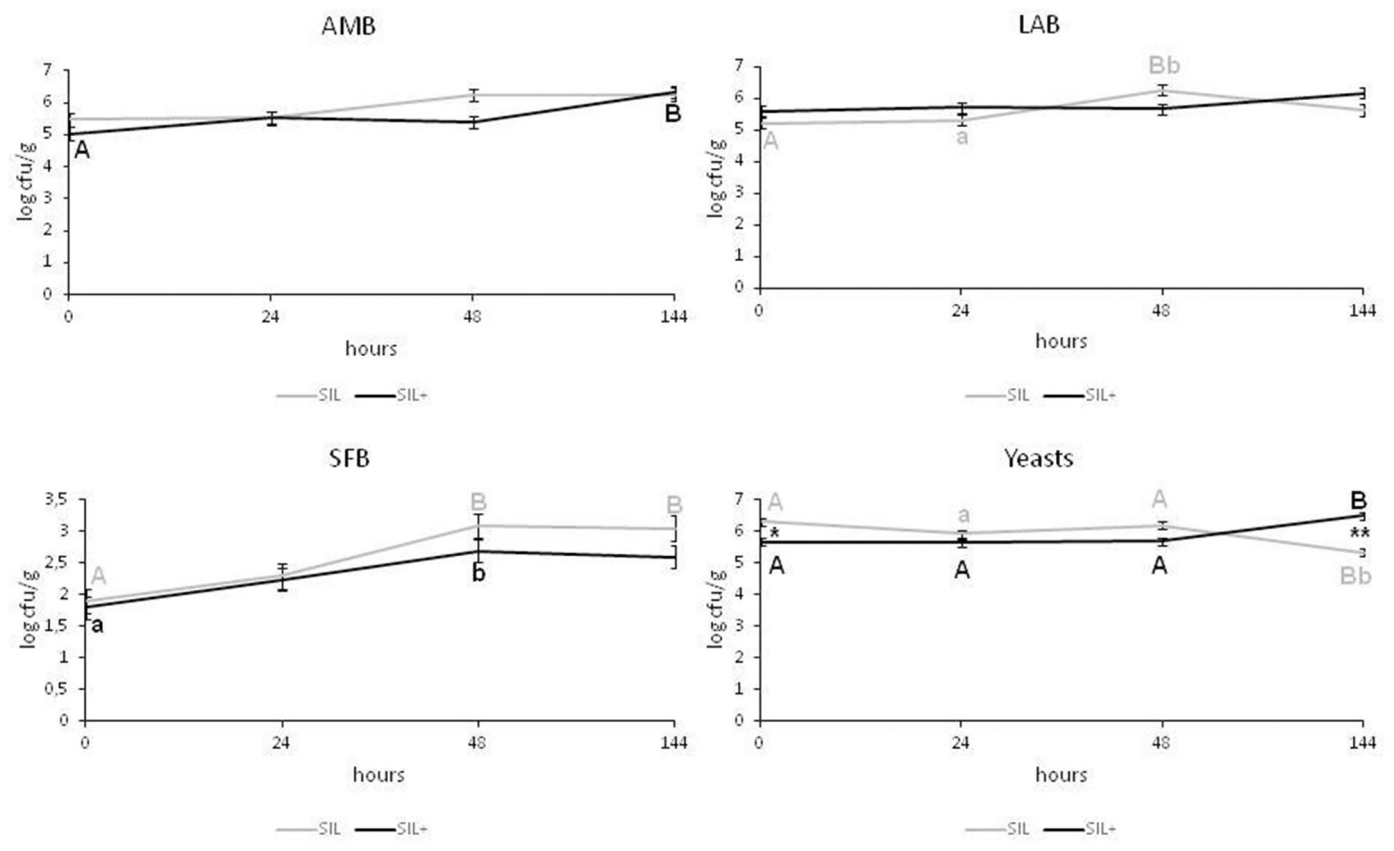
Microbiological characteristics of the silage with (SIL+) or without (SIL) *L. plantarum* 5BG inoculation. The microbial loads (log cfu/g) of total aerobic mesophilic bacteria (AMB), lactic acid bacteria (LAB), and spore-forming bacteria (SFB) and yeasts are reported. Values of each time point are the means of three replicates ± standard error (error bars). Different letters of the same color show statistical differences among time (A, B = *P* < 0.01; a, b = *P* < 0.05); *, ** show statistical differences between groups at the same time (*P < 0.05; **P < 0.01).

In SIL (Figure 2) the AMB counts did not vary significantly over time, while in SIL+ the values were significantly lower at the opening of the silage (0 h) (5 ± 0.04 log cfu/g) than at 144 h (*P* < 0.01). However, after 6 d (144 h) both silages presented equal values (6.3 log cfu/g). SIL+ showed LAB population ranging from 5.60 ± 0.15 log cfu/g (0 h) to 6.17 ± 0.15 log cfu/g (144 h) (*P* > 0.05), and both silages did not show significant differences at the same times.

At the opening of the silages and in all experimental times, SFB showed no differences between SIL and SIL+ (*P* > 0.05); however, they increased in SIL (P <0.01) and SIL+ (*P* < 0.05) after 48 h and then remained constant in both experimental trials.

After opening, the SIL yeast population was higher than SIL+ (5.66 ± 0.1 log cfu/g) (*P* < 0.05), but after 6 d yeasts increased in SIL+ reaching values (6.5 ± 0.19 log cfu/g) higher than SIL (*P* < 0.01).

Moreover, enterobacteria and *C. perfringens* were present in GP, but both were absent in silage; while molds and *Ls. monocytogenes* were absent in both GP and silages.

### Identification and Quantification of Phenolic Compounds and Antioxidant Activity

**Figure 3:**
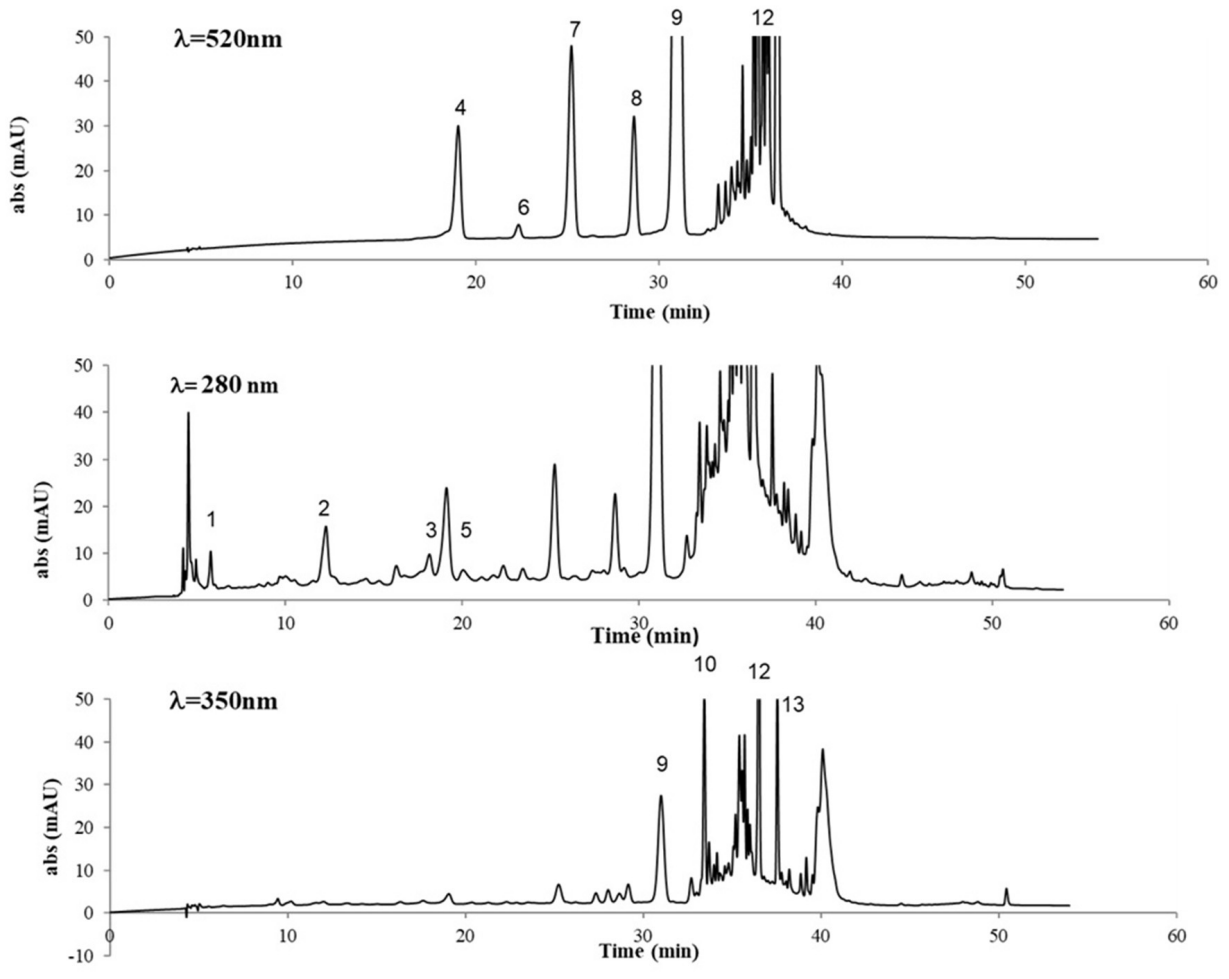
HPLC separation of phenolic compounds in GP extract (*V. vinifera* L., cv Nero di Troia), at λ = 520, 280, and 350 nm. For peak assignment, see Table 2.

The chromatographic profiles of GP extract at λ = 520, 280, and 350 nm are shown in Figure 3 and the identified compounds are listed in Table 2. The analytical profile of SIL and SIL+ extracts was comparable to that one of GP, only quantitative differences occurred.

**Table 2:** Peaks assignment of phenolic compounds extracted from grape pomace (*V. vinifera* L., cv Nero di Troia).

**No**.	**Compound**	**RT (min)**
1	Gallic acid	5.7
2	Catechin	12.3
3	Epicatechin	18.1
4	Delphinidin 3-*O*-glucoside	19
5	Syringic acid	20
6	Cyanidin 3-*O*-glucoside	22.3
7	Petunidin 3-*O*-glucoside	25.2
8	Peonidin 3-*O*-glucoside	28.6
9	Malvidin 3-*O*-glucoside	31
10	Rutin	33.4
11	Quercetin 3-*O*-glucoside	34.1
12	Malvidin 3-*O*-*p*-coumaroylglucoside *(t)*	36.5
13	Quercetin	37.5

*RT, Retention Time*.

*(t), Tentatively identified compound*.

The targeted HPLC analysis of GP extracts at λ = 520 nm revealed the presence of anthocyanins, with the typical five peaks of the monoglycosilated anthocyanins present in grape, according to the order delphinidin 3-*O*-glucoside, cyanidin 3-*O*-glucoside, petunidin 3-*O*-glucoside, peonidin 3-*O*-glucoside, and the predominant malvidin 3-*O*-glucoside, which were identified by comparison with reference compounds. Additionally, some peaks were detected with retention time higher than that of malvidin 3-*O*-glucoside, representing acylated anthocyanins, which were not assigned to specific structures, exclusively based on the UV-Vis spectra, except for the peak at RT= 36.5 being tentatively identified as malvidin 3-*O*-*p*-coumaroylglucoside (Figure 3).

Phenolic profile content of GP, SIL, and SIL+ is reported in Table 3. GP showed a total anthocyanins content higher than SIL (*P* < 0.05) and SIL+ (*P* < 0.01). SIL showed monoglycosilated anthocyanins, rutin, and epicatechin content lower than GP (*P* < 0.01) and the highest content of syringic acid (*P* < 0.01). SIL+ showed the lowest content of gallic acid, monoglycosilated anthocyanins, rutin, catechin, and epicatechin (*P* < 0.01) and the highest content of acylated anthocyanins (*P* < 0.01) and quercetin (*P* < 0.05). Moreover, SIL+ showed a catechin and epicatechin contents lower than SIL (*P* < 0.05) and GP (*P* < 0.01).

**Table 3:** Phenolic profile content (mg/g dry weight) in grape pomace (GP), after silage with (SIL+) or without (SIL) *L. plantarum* 5BG inoculation, quantified by HPLC analysis (*n* = 3).

	**Phenolic structure**	**Phenolic compounds**	**GP**	**SIL**	**SIL+**	**SEM**	***P*-value**
Non-flavonoids	Hydroxybenzoic acids	Gallic acid	0.08^A^	0.08^A^	0.06^B^	0.002	0.0006
		Syringic acid	0.04^B^	0.07^A^	0.04^B^	0.003	0.0013
Flavonoids	Anthocyanins	Monoglycosilated	2.53^A^	2.02^B^	1.23^C^	0.04	<0.0001
		Acylated	2.78^B^	2.70^B^	3.40^A^	0.09	0.0034
		Total	5.32^Aa^	4.72^b^	4.63^B^	0.11	0.0092
	Flavonols (as rutin eq.)	Rutin	0.32^A^	0.10^B^	0.02^C^	0.005	<0.0001
		Quercetin	0.17^b^	0.17^b^	0.27 ^a^	0.03	0.0442
	Flavanols (as catechin eq.)	Catechin	0.66^Aa^	0.42^b^	0.13^Bc^	0.04	0.0048
		Epicatechin	0.59^A^	0.39^B^	0.12^C^	0.02	<0.0001

*Different letters in the same line show statistical differences: A, B, C = P <0.01; a, b, c = P <0.05*.

*GP, grape pomace; SIL, grape pomace after silage; SIL+, grape pomace after silage with L. plantarum 5BG; SEM, mean standard error*.

Table 4 reports the total phenol content (TPC) and the antioxidant activity of GP, SIL, and SIL+. The TPC in SIL+ was lower than SIL (*P* < 0.01). The lowest ORAC activity was measured in SIL+ (*P* < 0.01), although no statistical differences were observed between GP, SIL, and SIL+ about TEAC activity (*P* > 0.05).

**Table 4:** Total phenol content (TPC) and antioxidant activity (by TEAC and ORAC assays) in grape pomace (GP), and after silage with (SIL+) or without (SIL) *L. plantarum* 5BG (per g dry weight).

	**GP**	**SIL**	**SIL+**	**SEM**	***P*-value**
TPC (mg GAE/g)	17.99^AB^	19.88^A^	16.60^B^	0.65	0.0063
TEAC (μmol TE/g)	140.82	134.85	121.92	10.06	0.4248
ORAC (μmol TE/g)	219.97^A^	218.36^A^	154.23^B^	8.78	0.0062

*Different letters in the same line show statistical differences: A, B = P <0.01*.

*GP, grape pomace; SIL, grape pomace after silage; SIL+, grape pomace after silage with L. plantarum 5BG; SEM, mean standard error*.

### *In vitro* Digestion and Gas Production

Table 5 reports the results of apparent percentage of disappearance after *in vitro* digestion. SIL+ showed the highest DM, OM, NDF, CP, EE, and NFC disappearance (*P* < 0.01). Moreover, SIL showed higher DM, OM, NDF, CP, EE, and NFC percentage of disappearance compared to GP (*P* < 0.01).

**Table 5:** Percentage of disappearance after *in vitro* digestion in grape pomace (GP), after silage with (SIL+) or without (SIL) *L. plantarum* 5BG.

	**GP**	**SIL**	**SIL+**	**SEM**	***P*-value**
DM (%)	39.5^C^	41.1^B^	43^A^	2.06	<0.0001
OM (%)	40.2^C^	41.3^B^	42.8^A^	2.08	<0.0001
NDF (%)	37.2^C^	40^B^	41.7^A^	1.83	<0.0001
ADF (%)	34.2	34	35.1	2.25	<0.0001
CP (%)	39.8^B^	39.3^B^	40.5^A^	0.77	<0.0001
EE (%)	29.5^C^	31.2^B^	32.9^A^	3.31	<0.0001
NFC (%)	51.8^C^	55^B^	58.1^A^	3.67	<0.0001

*Different letters in the same line show statistical differences: A, B, C = P <0.01*.

*DM, dry matter; OM, organic matter; NDF, neutral detergent fiber; ADF, acid detergent fiber; CP, crude protein; EE, ether extract; NFC, non-fiber carbohydrate; GP, grape pomace; SIL, grape pomace after silage; SIL+, grape pomace after silage with L. plantarum 5BG; SEM, mean standard error*.

*In vitro* gas production results are reported in Table 6. SIL+ showed the lowest propionic (*P* < 0.05) (*P* < 0.01) *in vitro* production and the highest butyric, methane (*P* < 0.01) and nitrogen (*P* < 0.05) *in vitro* production, although the total gas production and the total VFA production did not show differences among treatments (*P* > 0.05).

**Table 6:** *In vitro* gas production in grape pomace (GP), after silage with (SIL+) or without (SIL) *L. plantarum* 5BG.

	**GP**	**SIL**	**SIL+**	**SEM**	***P*-value**
Total gas production (mL)	19.8	19.5	20.1	0.36	0.2002
Total VFA (mmol/L)	91.3	90.1	91.0	0.36	0.3621
Acetic (mmol/L)	47.6	46.6	45.9	0.33	0.4052
Propionic (mmol/L)	28.3^a^	28.0^a^	26.9^b^	0.48	0.0254
Butyric (mmol/L)	14.3^B^	14.8^B^	18.7^A^	0.46	<0.0001
Methane (mmol/L)	11.3^B^	10.7^B^	13.1^A^	0.21	<0.0001
Nitrogen (NH_3_-N) (mmol/L)	14.1^b^	14.9^b^	16.0^a^	0.30	0.0182

*Different letters in the same line show statistical differences: A, B = P <0.01; a, b = P <0.05*.

*VFA, volatile fatty acids; GP, grape pomace; SIL, grape pomace after silage; SIL+, grape pomace after silage with L. plantarum 5BG; SEM, mean standard error*.

## Discussion

### Chemical Composition Characterization and Ensiling Effects

Feed digestibility is an important factor in the nutritional efficiency assessment of a ration. Among the three experimental treatments (GP, SIL, and SIL+), DM, OM, CP, EE, ash, NDF, ADF, and NFC values are often not consistent with those reported in the literature. For example, Spanghero et al. (38) observed slightly higher content of DM in Italian red and white grape pomace, but lower in the Californian red one. On the other hand, in both grape pomaces investigated, they observed similar values of OM and CP, higher values of EE and ADF and lower values of NDF. Massaro Junior et al. (39), studying South African red grape pomace, observed lower DM content, similar OM and NDF content, and higher EE, CP, and ADF content. Nevertheless, the high variability in the GP chemical composition depends on different factors such as the type of grape, grape maturity, wine production methods, components proportions (seeds and pulp), harvest methods (8, 38, 40–42). For these reasons it is difficult to compare values and results among different studies. Some authors stated that grape pomace DM should be >250 g/kg to obtain a good quality silage; some others defined an interval between 280 and 400 g/kg of DM to avoid high nutrient losses (39). The GP used in our experiment for ensiling showed a suitable DM content and the nutrient losses were almost null. Similarly, some authors observed no change in DM, CP, and ash after anaerobic storage (14). It can be supposed that this lack of differences can be due to the short storage time (43), and that ensiling both with and without *L. plantarum* 5BG addition had no great effect on grape pomace nutritional composition. Results showed that pH decreased with ensiling, and it decreased even more when *L. plantarum* 5BG was added. There are different approaches to the suitability of pH values for silage: some authors defined pH value 4.2 as the upper threshold for a positive assessment of a silage, while other researchers stated that the final pH is not important; what matters is the decreasing rate, as this parameter is more important in inhibiting a secondary fermentation occurrence (39, 44). The decrease of pH values during the ensiling process can be related to fermentation activities involving NFC consumption (39) but also WSC (14).

The results of the present work are consistent with this knowledge. LAB fermentation activities lead to the organic acid production with consequent pH reduction (19). Probably, the strain addition improved the fermentation processes inducing a lower concentration of NFC and WSC, and lower pH values. This result could be due to an efficient homofermentative lactic acid production promoted by the LAB strain (19).

The ensiling process reduced the grape pomace ME, but the *L. plantarum* 5BG addition seemed to enhance it. Similarly, Alipour and Rouzbehan (14) reported that ensiling reduced the grape pomace ME, probably due to a reduction of some nutrient availability and the incapacity of this storage process to reduce all the tannins and their negative effects on digestibility. Probably, the *L. plantarum* 5BG was able to increase grape pomace ME throughout a greater reduction of phenolic substances and consequently higher digestibility.

### Microbiological Quality

Plant-associated microbiota is composed of bacteria, including LAB, *Bacillus* sp., *Clostridium* sp., and coliform bacteria, as well as yeasts and molds. In general, a LAB number is considered a fundamental factor in predicting the suitability for the silage fermentation and in evaluating the need to apply the bacterial inoculants at ensiling. In fact, when the LAB number exceeds 5 log cfu/g, the silage can be well preserved. On the contrary, the low LAB number and the high number of harmful microorganisms could indicate the need to control the fermentation of the silage by LAB inoculation (18). The proliferation of undesirable microorganisms can cause direct DM and nutrients losses, but also indirect issues, due to reduced palatability and the risk of negative effects on the animal performance and health (16). In this current work, the epiphytic microbiota in GP was evaluated, and decided to proceed with the inoculation with *L. plantarum* 5BG, although high LAB counts were observed (ca. 5.9 log cfu/g). During ensiling, the role of LAB is to decrease pH by converting carbohydrates into organic acids which help to preserve the silage. Moreover, LAB inhibits the growth of undesirable microorganisms by competing for nutrients and synthesizing antimicrobial and antifungal agents (45). Different LAB species synthesize some metabolites, such as bacteriocins, hydrogen peroxide, and organic acids that inhibit the growth of pathogenic and spoilage bacteria, yeast, and molds (46). *L. plantarum* 5BG, used in our study as a grape pomace inoculant, was able to produce antimicrobial compounds and organic acids responsible for antifungal properties (47).

The results of the current work showed that the LAB number present in both silages (SIL and SIL+) can guarantee the quality of the silage. In fact, *Enterobacteriaceae* and *C. perfringens* were absent in the silages and SFB were in low number. After ensiling, both LAB and yeast populations were still high in number probably due to the carbon source quantities in GP that were suitable in supporting microbial growth. As observed in this study (Table 1), after ensiling, NCF decreased significantly probably due to their use by microorganisms. Furthermore, after ensiling, the tannin precursors decreased; therefore, a reduction of their inhibitory effect on the bacterial population could be hypothesized (21).

Yeast counts in GP were higher than the reported values in other silage crops (18, 48) and similar to those found in grape marc at the beginning of storage (49), while, after opening, yeasts were lower in SIL+ than in untreated and acidified marc studied by Maragkoudakis et al. (49). Probably, the yeast number decreased as results of pH reduction and antifungal activity of *L. plantarum* 5BG (47). During air exposure, yeast population remained substantially stable, however, as it is known, yeasts can cause aerobic deterioration and reduce the nutritional value of the silage (18). Therefore, further research is needed to improve yeast control.

### HPLC Analysis: Anthocyanins and Other Phenolic Compounds

While the silages, with or without the *L. plantarum* 5BG addition, did not show effects on the anthocyanins chromatographic profile (Figure 3), it revealed evidence of change in the anthocyanins concentration. In fact, ensiling decreased total anthocyanins content by 12%, whereas monoglycosylated anthocyanins content decreased much more (by 20%). Ensiling with *L. plantarum* 5BG addition led to a more significant reduction of monoglycosilated anthocyanins content than GP (reduction of 52%); comparing the two silage techniques, a 40% reduction occurred with LAB strain inoculation than without any supplement. The acylated anthocyanins content increased only in SIL+, reaching values of almost 20 and 25% higher than GP and SIL, respectively.

All these results point to the conclusion that the silage processes (with or without LAB strain inoculation) lead to an overall decreasing trend in the total anthocyanins content. On the other hand, acylated anthocyanins increased, exclusively with the addition of *L. plantarum* 5BG. Thus, it can be hypothesized that, during silage with inoculated LAB strain, monoglycosilated anthocyanins undergo acylation by acyltransferases of bacterial origin (50).

The total amount of anthocyanins found in GP (cv Nero di Troia) was within the range reported in other research (51–53), or to grape skin of the same cultivar Nero di Troia, limited to monoglycosilated anthocyanins (54).

The represented phenolic acids in the samples were the hydroxybenzoic acids, i.e., gallic and syringic acids. Gallic acid content did not change during silage (SIL) but decreased in SIL+. Syringic acid content was higher in SIL. As grape pomace is a highly oxidized product, syringic acid is supposed to be formed through oxidative degradation of malvidin 3-*O*-glucoside (55). The value we found in GP was consistent with previous reports for different grape cultivar pomace (56). With silage, a degradative oxidation of (monoglycosilated) anthocyanins occurred (with a 20% decrease), producing syringic acid from the degradation of malvidin 3-*O*-glucoside, possibly also from microbiological intervention. When GP was silaged with the addition of *L. plantarum* 5BG, monoglycosilated anthocyanins were further degraded (more than 50%) compared to GP sample, as oxidation of phenolic compounds can be supported by laccase from LAB origin (57). Conversely, syringic acid content did not change; the intervention of the strain 5BG could lead to a different metabolic fate of syringic acid.

Among flavonols, in GP rutin was predominant, followed by quercetin. In SIL samples, rutin content dropped (72% decrease), as well as in SIL+ samples (94% decrease). Moreover, the reduction in SIL+ was remarkable (78% reduction), even when compared to SIL. On the contrary, quercetin tended to increase in SIL+, being concomitant with the rutin decrease. This result was expected as quercetin is the aglycone which is released after rutin hydrolysis.

Catechin and epicatechin amounts occurred in GP samples after anthocyanins. The total flavanol content (catechin and epicatechin) was in the same range as reported in grape by-products from different red grape varieties (52, 58). The catechin was predominant over epicatechin, as usually reported, except in a few grape cultivars (59, 60).

Flavanols (catechin and epicatechin) content in SIL and SIL+ decreased by ~36% and ~80% of the value in GP, respectively. The reduction of flavanol content could be a positive aspect, as catechin and epicatechin are the precursor compounds of condensed tannins, which, as described in the published literature, tend to influence the digestibility of the feeding ration (61, 62). When GP has been used for feeding purposes (13), the reported content of epicatechin was 0.11 mg g^−1^, comparable to the content found in our experiments after ensiling with *L. plantarum* 5BG addition.

It has been reported already that the anaerobic storage of a plant matrix leads to a decrease of polyphenolic content (43). In ensiled grape pomace in particular, Alipour and Rouzbehan (14) found a reduction of total phenols and tannins. Moreover, it has been shown that storage increased the polymerization of existing tannins into higher oligomers (63). Thus, it can be hypothesized that polymerization occurs at the expenses of tannins precursors (flavanols). *L. plantarum* could be more efficient to promote polymerization than the existing microorganisms in the silage.

### Hydrophilic Antioxidants: Phenols and Antioxidant Capacity

The phenols content in GP (cv Nero di Troia) was in the same range as in past publications, both in different Italian red grape cultivars (53), and international cultivars (64), also used for feeding cows (13, 53).

With ensiling, the phenol content was statistically lower in SIL+. The same trend was shown for the antioxidant capacity: silage, with or without *L. plantarum* 5BG addition, reduced the total phenol content and, consequently, the antioxidant capacity of the samples.

The antioxidant capacity in GP, assessed by TEAC assay, was similar to that reported by González-Paramás et al. (58), but lower than Rockenbach et al. (65). The ORAC value in our experiments revealed the same trend reported by Yilmaz and Toledo (64) in international grape varieties, even if they used β-phycoerithrin as fluorescent marker instead of fluorescein.

### Apparent Percentage of Disappearance After *in vitro* Digestion and Gas Production

Rumen digestibility represents an important factor to define feeds nutrients productive efficiency and animals' environmental impact. Therefore, the evaluation of the inclusion of innovative food on rumen digestibility plays a key role for in field application (66). Ensiling did not affect most of the chemical constituents, both with and without *L. plantarum* 5BG inclusion, but a more significant effect on *in vitro* digestibility was found. Ensiling increased the DM, OM, NDF, CP, EE, and NFC percentage of disappearance, and these results were even improved by *L. plantarum* 5BG inoculation. Past publications reported that ensiling GP released secondary compounds as tannins (38) and that they may negatively affect digestibility, particularly because they can bind crude proteins, minerals, and carbohydrates (41, 67). The current study showed that ensiling reduced the GP flavanols (tannins precursors) content and that the *L. plantarum* 5BG addition reduced even more their concentration. Similarly, other researchers reported that anaerobic storage like ensiling can be used to reduce tannins content of grape pomace (43, 68). It is known that *L. plantarum* is a LAB species able to degrade phenolic compounds, but the metabolic pathways of their degradation in LAB have not been completely described (21). Furthermore, many factors can influence the results of a LAB addition, such as the characteristics of raw materials, epiphytic microbiota, and the silage management. A recent work (20) investigated the biotransformation of phenolic compounds in sainfoin silage inoculated with *L. plantarum* and observed the reduction of flavanols (catechin and epicatechin). The flavanols content reduction can explain digestibility differences found in this study among SIL, SIL+, and GP (61, 62). The antioxidant activity of these secondary compounds (tannins, anthocyanins, etc.) can be exerted also on the rumen microbial activity reducing and/or inhibiting some of them and, consequently, reducing the digestive capability. It is well known that *Lactobacillus* addition, apart from accelerating lactic acid production and inhibiting detrimental microorganisms' growth, may also further control microorganism population conferring antimicrobial properties to silage and outcompeting them for free sugars (69, 70). On the other hand, it is inferred that *Lactobacillus* addition improves digestibility likely throughout the reduction of all phenolic compounds that may inhibit microbial activity (71, 72). Total gas production values for GP were similar to those reported by other authors (38), and they increased in SIL+. Differently from what was reported by some authors (14), the reduction in flavanols content in SIL+ could be the main cause of the increase in gas production during digestion. The total volatile fatty acids production did not change, but there was an increase in butyric acid and a slightly decrease in propionic acid in SIL+ compared to both GP and SIL.

The substrate for the production of butyric acid is dietary fiber, which undergoes different metabolic pathways as a result of bacterial fermentation, starting from glucose and, consequently, carbohydrates availability (73). Ensiling reduced the NFC content, likely because the latter represent the first suitable substrate for the growth of the microbiota, as also confirmed by the *L. plantarum* 5BG addition. Moreover, NFC digestibility increased with ensiling probably due to the lower total phenol content and flavanols (condensed tannins precursors), resulting in a higher and faster butyric acid production and thus a lower and slower propionic acid production.

Ammonia nitrogen represents one of the major sources for microbial growth and microbial protein synthesis in the rumen (32, 74) and its production rate is strictly linked to the dietary rumen protein degradation (75). The increased ammonia nitrogen values observed in *L. plantarum*-added silage probably could be due to the increased protein digestibility, considering that high antioxidant component inclusion in feed tends to reduce the ammonia nitrogen concentration (75). This result shows how ensiling processes can give more nitrogen availability and digestibility with a consequent higher nutrient availability for microbial populations. In fact, some authors reported that ammonia concentration, a key metabolite for nitrogen production, decreased due to a lower protein degradation in the feed and a lower microbial protein synthesis (32, 75).

The CH_4_ production tended to be higher in the SIL+ compared to the other treatments. It seems that ensiling with *L. plantarum* 5BG increased its production. Methane production depends not only on the available fermentable substrate amount but also to the proportion of VFA produced during the fermentation (76). Also condensed tannins content has been reported to correlate negatively with CH_4_ production (77). Tannins have both the ability to reduce protein and carbohydrate availability for rumen microorganism fermentation, complexing them (61), and the capacity to inhibit bacteria, protozoas, and archeans, activity (62). Furthermore, tannins may inhibit the growth or activity of rumen methanogen and protozoa through bactericidal or bacteriostatic activities (78), most likely through binding proteins and enzymes of the microbial cells (79). Although the lower content of NFC, the lower phenols content in the SIL+ probably had a synergic effect combining more substrate availability for fermentation and lower phenols inhibition of fermentation.

## Conclusion

The results of this study suggested GP as an alternative ingredient in animal feeding. The nutrient content of GP did not show important changes with ensiling, except for the reduction of NFC content, probably because of its role in fermentation kinetics. In addition, the level of condensed tannins precursors (flavanols) was reduced by the ensiling process. This was more effective if the *L. plantarum* 5BG was added to the silage. Moreover, total phenol content and antioxidant activity were both reduced by strain 5BG addition. These combined observations are consistent with the *in vitro* digestibility of the ensiled grape pomace, particularly when *L. plantarum* 5BG was added. In fact, all parameters investigated show increased percentage of disappearance, confirming a better *in vitro* digestibility of GP if ensiled with *L. plantarum* 5BG. Furthermore, ensiling with *L. plantarum* 5BG addition was able to affect *in vitro* rumen fermentation patterns and methane *in vitro* production. Although total volatile fatty acids production did not show differences, butyric acid and methane production slightly increased. In conclusion, ensiled grape pomace can represent an important source of digestible nutrient for ruminants and SIL+ may be an alternative feed. However, additional research demonstrating the *in vivo* utilization, or at least further advanced *in vitro* techniques are needed.

## Data Availability Statement

The raw data supporting the conclusions of this article will be made available by the authors, without undue reservation.

## Ethics Statement

The animal study was reviewed and approved by Ethics Committee for Animal Testing–CESA (process number 2-X/17) of the Department of Veterinary Medicine of the University of Bari.

## Author Contributions

PDB: investigation, formal analysis, writing-original draft, and writing-review and editing. AM: conceptualization, investigation, data curation, formal analysis, writing-original draft, and writing-review and editing. CA: investigation. PDP: conceptualization, investigation, funding acquisition, resources, project administration, and writing-review and editing. FB: investigation, visualization, supervision, writing-original draft, and writing-review and editing. All authors contributed to the article and approved the submitted version.

## Funding

This research was funded in part by the CNR-DiSBA project NutrAge (FOE-2019, DSB.AD004.271).

## Conflict of Interest

The authors declare that the research was conducted in the absence of any commercial or financial relationships that could be construed as a potential conflict of interest.

## Publisher's Note

All claims expressed in this article are solely those of the authors and do not necessarily represent those of their affiliated organizations, or those of the publisher, the editors and the reviewers. Any product that may be evaluated in this article, or claim that may be made by its manufacturer, is not guaranteed or endorsed by the publisher.
